# Lactate-mediated Fascin protrusions promote cell adhesion and migration in cervical cancer

**DOI:** 10.7150/thno.83938

**Published:** 2023-04-17

**Authors:** Xiao Han, Shujuan Du, Xiaoting Chen, Xuehua Min, Zhongwei Dong, Yuyan Wang, Caixia Zhu, Fang Wei, Shujun Gao, Qiliang Cai

**Affiliations:** 1 Center of Diagnosis and Treatment For Cervical & Uterine Cavity Disease, Obstetrics and Gynecology Hospital of Fudan University, Shanghai Key Laboratory of Female Reproductive Endocrine-Related Disease, & MOE/NHC/CAMS Key Laboratory of Medical Molecular Virology, Shanghai Institute of Infections Disease and Biosecurity, Shanghai Frontiers Science Center of Pathogenic Microorganisms and Infection, School of Basic Medical Sciences, Shanghai Medical College, Fudan University, Shanghai 200032, P. R. China.; 2 ShengYushou Center of Cell Biology and Immunology, School of Life Sciences and Biotechnology, Shanghai Jiao Tong University, Shanghai 200240, P. R. China.

**Keywords:** Lactate, LA antagonist, β-catenin/fascin, Cervical Cancer

## Abstract

**Background:** Lactate is associated with the poor prognosis of many human malignancies. Cervical cancer, one of main causes of women mortality worldwide, is aggressive and absent of effective pharmacological treatment, and its underlying mechanisms of progression remain elusive.

**Methods:** The regulation of β-catenin to fascin protrusion formation upon acidic lactate (Lactic acid [LA]) stimulation was evaluated through in β-catenin or fascin deficiency cell line models by immunofluorescence assays, and subcellular fractionation. The effect of β-catenin and fascin relocation by LA and its antagonist were evaluated by immunohistochemistry assay in patient tissues and mouse tumor xenograft model. Trypsin digestion, Transwell assay, cell proliferation *in vitro* was performed to explore the role of LA in the cell growth, adhesion and migration.

**Results:** Low concentration of LA significantly promotes cytoskeleton remodeling via `protrusion formation to increase cell adhesion and migration. Mechanistically, upon LA stimulation, β-catenin diffuses from the cytoplasmic membrane into the nucleus, which in turn induces fascin nuclear-cytoplasm redistribution to the protrusion compartment. Moreover, the antagonist of LA sufficiently blocks the LA-mediated β-catenin nuclear import, fascin nuclear export, and the growth and invasion of cervical cancer cells *in vitro* and* in vivo* using a murine xenograft model.

**Conclusions:** This study uncovers β-catenin-fascin axis as a key signal in response to extracellular lactate and indicates that antagonist of LA may serve as a potential clinical intervention for cancer development.

## Introduction

Cell metabolic reprogramming has been recognized as a major source of cellular energy and mass, and commonly occurs in a variety of malignancies 1. In tumor cells, coupled with tricarboxylic acid (TCA) and oxidative phosphorylation, both glycolysis and glutaminolysis lead to the production of lactate by cytoplasmic enzyme lactate dehydrogenase (LDH-A)-mediated conversion of pyruvate 2. The physiological roles of lactate and its acidic form (lactic acid [LA]) have been controversial since their discovery in biological tissues 3. In the past decade, increasing evidence has shown that lactate plays multiple roles in cell homeostasis by serving as a metabolic fuel, buffering agent, or a signaling molecule. Owing to the robust transport of lactate across the plasma membrane by monocarboxylate transporters (MCT-1/4), and the participation with its receptor GPR81 (also known as hydroxycarboxylic acid receptor 1 [HCAR1]), the extracellular lactate has been widely used as a marker of intracellular NADH: NAD+ status for ATP production, and as biomarker in various diseases.

It has been documented that the ability of metastatic cancer cells to escape from the primary tumor, invade the surrounding tissue or circulatory system, and establish secondary tumors, is the main cause of poor prognosis of human malignancy. The dissemination of tumor cells requires cell migration, which is driven by the dynamic reorganization of the cytoskeleton 4, 5. Fascin, one of the main actin-bundling protein of the cytoskeleton reorganization, is essential for cross-linking flexible actin filaments into stiff and rigid bundles during pseudopodia, invadopodia, and cell adhesion 6-9. Fascin is low or absent in normal human adult epithelial cells, but is highly expressed in metastatic cancer cells. Increasing evidence has shown that elevated levels of fascin in many types of metastatic cells contribute to cancer cell migration and invasion during metastatic dissemination, and are correlated with clinically aggressive phenotypes, poor prognosis and overall survival 10-12. Additionally, several studies have demonstrated that β-catenin plays a key role in regulating and coordinating intracellular adhesion via ligation between the cytoskeleton and membrane 13-15, and the translocation of β-catenin into the nucleus up-regulates the transcriptional level of fascin 16, 17. Fascin inhibitors have been shown to efficiently block the migration of intratumoral dendritic cells and cancer growth in xenograft mouse model and clinical studies 8, 12, 18-20. High lactate levels circulating in the blood have often been shown to increase the risk of metastasis of many cancers 21-23, and our recent studies have shown that low concentrations of LA can trigger Epstein-Barr Virus (EBV)-immortalized B lymphoblastic cell adhesion by downregulating viral miRNA expression 24. However, whether lactate impairs attached (epithelial, non-suspended B cells) cancer cell metastasis, particularly with respect to morphological changes and migration, and the underlying mechanisms are not yet fully elucidated.

Cervical cancer is one of the most common malignant tumors in women, and the leading cause of cancer-related mortality in women worldwide 25. Persistent high-risk HPV (human papillomavirus) infection has been confirmed as an identifiable cause of cervical cancer 26-29. Although HPV-encoded E6 and E7 have been demonstrated as key oncoproteins, HPV infection alone is insufficient to cause cervical lesions 30. According to epidemiological research, the infection rate of sexually active women is up to 80% 31, 32. Fortunately, most HPV infections are self-limiting 33, 34, thereby the immune interaction between the host, virus, and microenvironmental factors may serve to resist HPV invasion and colonization, and facilitate viral clearance 35, and only a small percentage of persons go on to develop a persistent high-risk HPV infection that eventually leads to cervical cancer 36, 37. The different outcomes of HPV infection suggest that other complicated factors have vital influences on HPV-mediated carcinogenesis, the identification of which will likely provide effective therapeutic targets for cervical cancer.

The vaginal microbiome, analogous with the intestinal microbiome, is the dynamic homeostatic system of the vagina, changing with the menstrual cycle, age, and many other factors 38, 39. Several studies have shown that the imbalance of the vaginal *Lactobacillus* (a probiotics) microenvironment is closely related to HPV clearance obstacles and cancer progression 40, 41. Increasing data show that the vagina flora can be divided into five types of *Lactobacillus crispatus*, *L. gasseri*, *L. inners*, *L. jensenii* and a variety of strict anaerobic bacteria (*BV*) 42. Interestingly, both *L. crispatus* and *L. gasseri* have been identified to produce two isomeric forms of lactate, while *L. inners* and* L. jensenii* mainly produce only one form of LA, respectively 40. Therefore, two isomeric forms of LA are crucial constituents of vaginal microecology 43, and seem to exert two-opposing effects due to different phenomena. For example, the isoform of LA produced by the probiotic *Lactobacillus sp*. provides an extremely low pH (pH = 4.5) in the cervicovaginal environment, which is capable of defending against various urogenital infections 39, 44-47. Thus, a reduction of *Lactobacillus* will increase the risk of cervical lesions 44, 48, 49, while as a main product of glycolytic route, lactate accumulation has been shown to be closely associated with increased metastasis and poor prognosis of patients with cervical cancer 50, 51. In contrast, the isoform of LA secreted from *Lactobacillus* species (e.g., *L. crispatus*, *L. gasseri* and *L. jensenii*) has been reported to be a protective factor against HPV infection and cervical lesions 40, 43. It is worthy to pay attention that although the two isoforms of LA from the supernatant of vaginal *Lactobacillus* could play different roles in cervical cancer progression, how LA affects cancer progress and their underlying mechanisms remain largely unknown.

In this report, using cervical cancer cells as a model, we demonstrated that low concentrations of LA can efficiently induce cell protrusion formation during cytoskeleton remodeling, which is linked to increased tumor cell adhesion and migration *in vitro*. Upon lactate stimulation, β-catenin loses membrane fixation and diffuses into the nucleus, whereas fascin translocates to the cytoplasm and co-localizes at the LA-induced protrusion compartment. Moreover, β-catenin knockout significantly inhibits the ability of LA-mediated fascin translocation and protrusion formation. Morphological observation and immunohistochemistry analysis from different cervical patient tissues and a murine xenograft tumor model, further revealed that LA-mediated nuclear-cytoplasmic redistribution between β-catenin and fascin is correlated with the growth and invasion of cervical cancers. Interestingly, the antagonist of LA could sufficiently block LA-mediated effects on the β-catenin membrane anchor and fascin-mediated protrusion formation *in vitro* and *in vivo*. Overall, our findings provide a potential strategy for the early intervention of cancer development.

## Materials and Methods

**Human Subjects −** Cervical squamous cell carcinoma (CC, n=5), high-grade squamous intra-epithelial lesion (HSIL, n=5), and normal cervical (NIML, n=5) tissues were collected by colposcopy biopsy from patients without any preoperative therapy in the Obstetrics and Gynecology Hospital affiliated to Fudan University, China from 1/1/2021 to 6/1/2021. The tumor stage of cervical cancer was identified according to surgical and pathological findings, and the International Federation of Gynecology and Obstetrics (FIGO) stage system. Ethical approval was obtained from the ethics committee of the Fudan University-affiliated Obstetrics and Gynecology Hospital. Written informed consent from patients were obtained.

**Cell Lines −** HeLa (isolated from female patient, American type culture collection, ATCC), CaSki (isolated from female patient, ATCC), and C33A (isolated from female patient, ATCC) were maintained in DMEM supplemented with 8% FBS and 1% Penicillin (Sangon Biotech) and Streptomycin (BBI Life Sciences). All cell lines were incubated at 37℃ in humidified environmental incubator with 5% CO_2_.

**Animal Ethics Statement and Husbandry −** The China Guide for the Care and Use of Laboratory Animals was followed in all animal research. All studies were approved and supervised by Fudan University's institutional animal care and use committee under Protocol ID 196086. Five-week-old female BALB/c nude (CAnN.Cg-*Foxn1*^nu^/Crl, 401) mice were purchased from Beijing Charles River Laboratory Animal Technology Co., Ltd (Beijing, China), and were housed in certified specific-pathogen-free or germ-free microisolator cages with no more than 5 mice per cage. Animals have unlimited access to sterile water and food throughout the research period.

**Antibodies and Reagents −** Antibodies against HPV16/18 E6 (sc-460, Santa Cruz), HPV18 E7 (sc-365035, Santa Cruz), HPV16 E7 (sc-6981, Santa Cruz), α-tubulin (Proteintech), β-catenin (#8480, cell signaling), Fascin (126772, Abcam), and GAPDH (Proteintech) were used according to the manufacturer's instruction. Protease inhibitors PMSF, Aprotinin, Leupeptin and Pepstatin A were purchased from Amresco Inc. Acidic Lactate (LA) was purchased from Sigma-Aldrich (cat. #27714) and D-LA was from TCI (cat. #10326-41-7).

**Immunofluorescence Analysis −** Immunofluorescence assays were performed as described previously with modification 52. Briefly, cells were harvested and fixed in 4% paraformaldehyde for 20 min in dark at room temperature, and then washed with PBS twice. The fixed cells were then washed three times with blocking buffer PBS containing 0.2% fish skin gelatin (G-7765; Sigma) and 0.02% TritonX-100, and permeabilized in blocking buffer, followed by primary and secondary antibodies (anti-mouse Alexa Fluor 594/488 and anti-Rabbit Alex Fluor 594/488 1:500) staining or phalloidin-conjugated with TRITC staining for cytoskeleton according to product instruction. Nucleus was stained with 4, 6-diamidino-2-phenylindole (DAPI), and coverslips were mounted with p-phenylenediamine. Cells were visualized with Leica SP8 confocal microscope. Cells with more than 5 protrusions that longer than 5 μm are defined as cells with robust protrusion.

**Trypsin digestion assay −** After treated with PBS, LA, LA-Na, and H^+^ for 24 hours and washed with PBS twice, CaSki cells were digested by trypsin for 4 min at 37ºC. The detached cells are washed by PBS twice. Cells remained on the flasks are defined as trypsin resistant cells (undigested cells) and were visualized with Evos 2000 to count undigested protrusion. After visualization, remained cells are digested twice to calculate total cell number. Detached cell proportion is calculated by detached cell number/ total cell number.

**Transwell Assay for Cell Migration −** Cell migration was assessed by Transwell cell culture chambers (8.0 μm, corning falcon, cat.3422), according to the manufacturer's protocol. 1 × 10^5^ HeLa cells or 1.5x10^5^ SiHa cells were suspended in 200 μl of serum-free medium and placed in a Transwell insert. The lower chamber was filled with 600 μl of medium containing 10% FBS. The cells were then incubated for 18 h at 37ºC in a humidified environmental incubator with 5% CO_2_, washed twice with PBS, fixed with 4% paraformaldehyde for 15 min and subsequently stained with 0.1% crystal violet for 10 min at room temperature. The cells that adhered to the upper surface of the chamber were carefully removed by wet cotton swabs, and those on the bottom surface of the membrane were imaged by Nikon fluorescence microscope. Three randomly selected fields were counted using Image J software.

**Cell Proliferation in Vitro −** Total of 0.5×10^5^ CaSki cells were seeded into 10 cm^2^ 6-well plate with 2 ml DMEM medium treated with 2.5 mM LA only or 2:1 ratio with A001, or PBS, and cultured at 37ºC for 5 days. The culture medium is replaced every 24 h. The cell numbers were counted using Vi-cell ^TM^XR (Beckman coulter) at different time points.

**Immunoblotting −** Immunoblot analyses were performed by using standard methods. Briefly, cells were harvested and lysed in the radioimmunoprecipitation assay (RIPA) buffer containing protease inhibitors (Sigma-Aldrich) and phosphatase inhibitors (Keygen, China). Proteins were separated by SDS-polyacrylamide gel electrophoresis gels and transferred to 0.45-mm nitrocellulose membrane (Millipore). The membrane was probed with the primary antibodies as indicated and appropriate IRDye-800CW-conjugated secondary antibodies, and scanned with an Odyssey Infrared scanner (Li-Cor Biosciences). The relative intensity is quantified by image J.

**Subcellular Fractionation −** Nuclear and cytoplasmic fractionation were performed by using Minute^TM^ Cytoplasmic and Nuclear Extraction Kit for Cells (Cat. #: SC-003) as described previously with modified^52^. Briefly, CaSki cells after treated with or without LA for 2 h were harvested and washed twice with ice-cold PBS followed by resuspending and lysis cell pellet in appropriate amounts of cytoplasmic extraction buffer for 5 min on ice, followed by 14,500 rpm for 5 min at 4˚C. The supernatant (cytoplasm protein) harvested and frozen at -80˚C for use. The nuclear pellets were washed twice with 0.5 ml cold PBS to reduce contamination of cytosolic proteins, resuspended in nuclear extraction buffer, and lysed on ice with every 1 min vortex 15 s for 4 times, followed by 14,500 rpm for 5 min at 4˚C. Supernatant (nuclear protein) was harvested and snap frozen for further use. The efficiency of cytoplasm and nuclear extraction were verified by immunoblotting with Lamin B1 and α-Tubulin antibodies, respectively.

**CRISPR/Cas9-mediated Knockout −** Fascin targeting sequence (sgRNA, 5'-CACCGGCCGGCCACTGGCTACACGC-3'), β-catenin targeting sequence (sgRNA, 5'-CACCGAAAATGGCAGTGCGTTTAGC-3'), and scramble control sequence (sgRNA, 5'-CACCGGTAGCGAACGTGTC CGGCGT-3') were designed according to Zhang Lab website: https://zlab.bio/guide-design-resources. Plasmids pLenti-CRISPR -Fascin, β-Catenin or ctrl was individually constructed by adding the annealed oligos into pLentiCRISPRv2 vector with digestion of restriction enzymes *Bsm*BI. Caski or SiHa cells were transient transfected with pLenti-CRISPRv2-fascin or Lenti-CRISPRv2-β-catenin using PEI 4000/jetOPTIMUS according to the manufacturer's instructions, followed by 48-h treatment with 1 μg/mL of puromycin at 24-h posttransfection. Immunoblot analysis with fascin and β-catenin antibodies were used to verify the efficiency of target gene knockout.

**Immunohistochemistry Assay −** Immunohistochemistry analyses of β-catenin, fascin, LDH-A, and CD34 were performed on deparaffinized, formalin-fixed tissue sections using an indirect immunoperoxidase method with an automated immunostainer as described previously 53.

**Tumor xenograft −** Five-week-old female BALB/c nude mice in similar weight were randomly divided into 4 groups. Each mouse (five mice/group) was individually injected subcutaneously with 10 million of SiHa cells (in 100 μl PBS) at flank region to achieve tumor model. The mice were individually subjected to intratumor administrate with 2.5 mM LA, A001, LA/A001 (2:1, equal pH as LA), or PBS in 50 μl volume at day 0, 2, 4 and 6 of 9-day post-inoculation. All mice were sacrificed at 19 days post-inoculation. Each tumor was excised and measured, and subjected to immunohistochemical staining for LDH-A, CD34, fascin and β-catenin.

## Results

### LA promotes protrusion formation in cervical cancer cells

**Figure 1:**
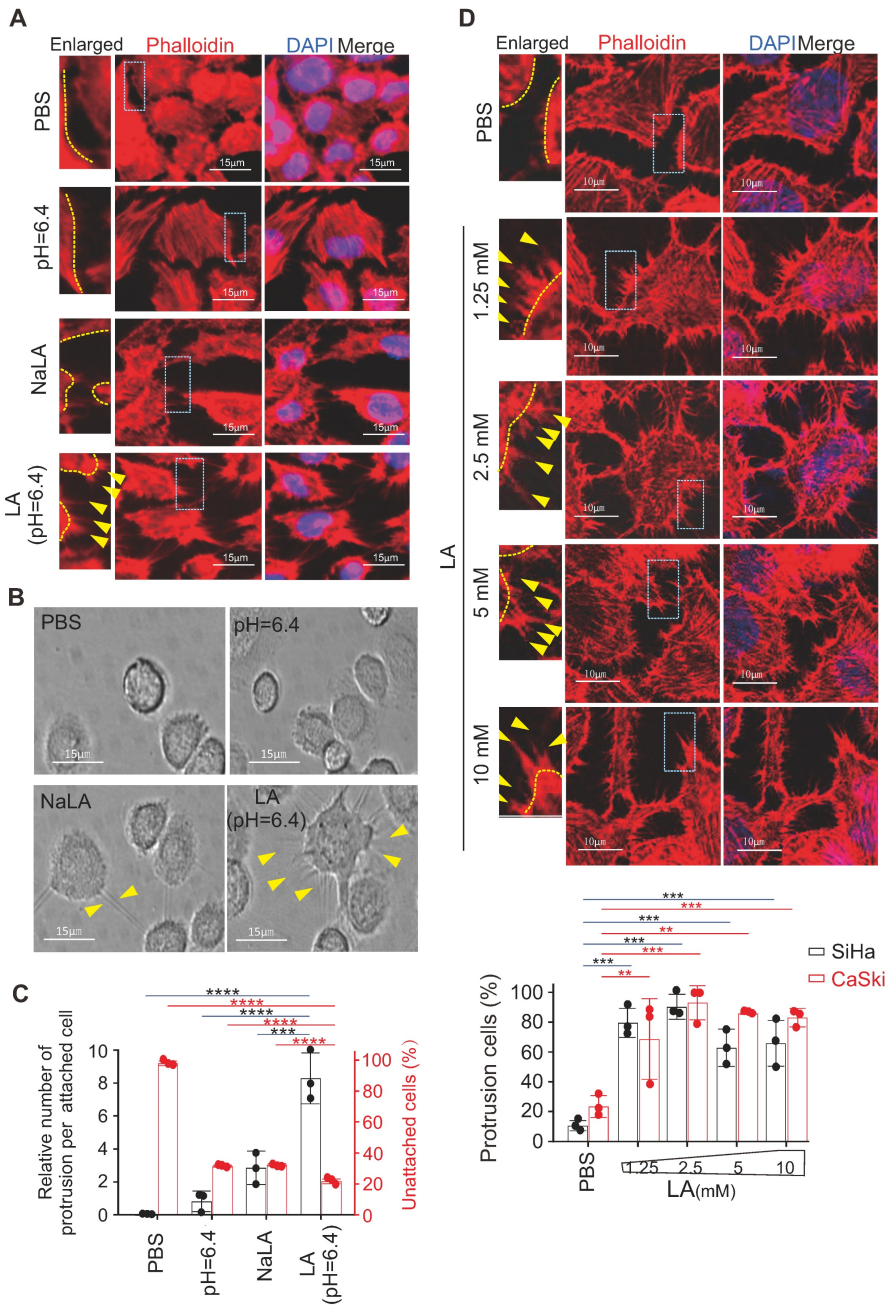
** Lactic acid enhances protrusion formation in cervical cancer cells.** (**A**) Lactic acid (LA) induces morphological changes in SiHa cells. Representative EVOS immunofluorescence microscopy photographs of cell morphology after treatment with 10 mM LA, LA-Na, PBS or medium at pH=6.4 for 24 h are shown. Cells were stained with phalloidin conjugated with TRITC. Nuclei are stained by DAPI. Yellow arrows in enlarged image indicates prolonged protrusions. (**B**) Representative microscopic photographs of CaSki cells with or without LA treatment. Yellow arrows indicating trypsin-resistant protrusions. (**C**) The proportion of attached cells with visible protrusions and the percentage of unattached cells after 4-min trypsin digestion from *panel B*. (**D**) Low concentrations of LA substantially induces protrusion formation in cervical cancer cells. Representative confocal microscopy photographs of SiHa cells treated with different dosage of LA (1.25, 2.5, 5, or 10 mM) or PBS for 24 h, were stained with phalloidin and DAPI as described in panel A. Yellow arrows indicates prolonged protrusions. *Bottom panels*, the percentage of cells with visible protrusions was calculated. Cells with more than 5 protrusions that longer than 5 ㎛ are defined as cells with robust protrusion. Data are presented as the mean±SD of three independent experiments. Asterisks indicate significant difference (*p < 0.05, **p < 0.01, and ***p < 0.001).

Our previous study showed that 10 mM LA induced a significant morphological change in EBV-immortalized B-lymphoblastic cells 24; however, whether and how LA influences attached epithelial (non-suspended B cell) cells remains unknown. Thus, using cervical cancer cells as a model, we attempted to individually treat different cervical cancer cell lines (including SiHa, HeLa and CaSki) with phosphate buffered saline (PBS), sodium lactate (NaLA), proton H+ (equal pH value), or LA for 24 h, followed by cytoskeleton staining with Phalloidin-conjugated dye and photographing to observe the morphology change. Interestingly, distinct from the paralleled controls, only LA dramatically enhanced protrusion formation in all of three cervical cancer cell lines (Figure 1A, bottom panels, supplementary Figure S1A). To answer whether LA does impair other type of cancer cells, we tested and also observed similar morphology change occurrences in hepatoma cell line Ad38 and gastric carcinoma cell lines CRL-5822 and AGS-Luc2, upon LA treatment (supplementary Figure S1B). As the cytoskeleton is important for cell adhesion, invasion, and metastasis, we first observed the attached morphology of CaSki cells after LA treatment, following by trypsin digestion. Consistently, we found great amount of undigested CaSki cells presented much more trypsin-resistant cell protrusions than that observed in the PBS-, proton H+- or NaLA-treated groups, albeit a few cell protrusions in the NaLA-treating group appeared (Figure 1B-C), and less digested cells were found in the LA-treated group (Figure 1C). To confirm this phenomenon, we repeated similar experiments using cervical cancer cell lines SiHa and CaSki with lower concentrations (1.25 mM, 2.5 mM, 5 mM, and 10 mM) of LA for 24 h. Intriguingly, we found that the lowest concentration (1.25 mM) of LA treatment was sufficient to induce conspicuous protrusion formation in both SiHa and CaSki cells (Figure 1D, middle and bottom panels), and exhibited significantly increasing amount compared to the PBS group (Figure 1D, bottom panels). These results indicate that LA is capable of promoting protrusion formation and cell adhesion in cervical cancer cells.

### Antagonist of LA blocks LA-induced protrusion formation *in vitro*

**Figure 2:**
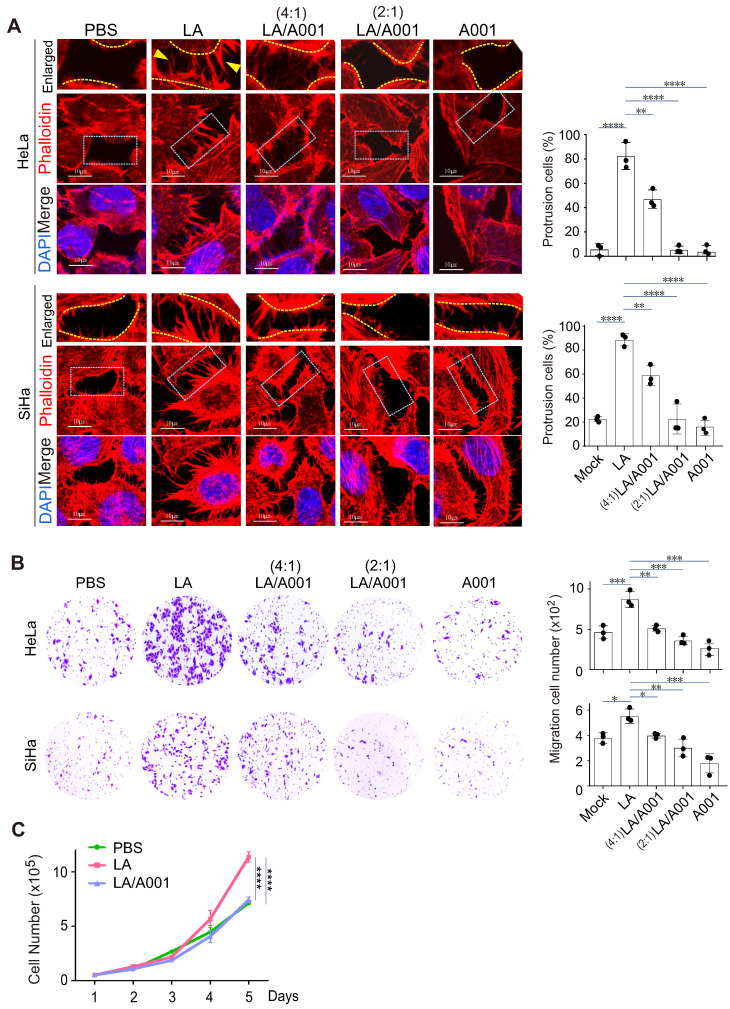
** Antagonist of LA significantly blocks LA-mediated malignant beha**vior. (**A**) Antagonist of LA significantly attenuated LA-mediated protrusion formation in both HeLa and SiHa cells. Cells treated with PBS, 2.5 mM A001, 2.5 mM LA only or different ratio with A001 (4:1, or 2:1) for 24 h were stained with phalloidin and DAPI as described previously. The percentage of cells with visible protrusions was calculated and shown on the *right panels*. Cells with more than 5 protrusions that longer than 5 ㎛are defined as cells with robust protrusion. Yellow arrows indicate prolonged protrusions. (**B**) The motility of HeLa /SiHa cells treated with LA, A001 or PBS as described in panel A was assessed by Transwell assays. Migrated cell number was calculated and shown on the right panels. (**C**) Proliferation of CaSki cells treated with 2.5 mM LA only or a 2:1 ratio with A001 or PBS for different time periods. Data are presented as the mean±SD of three independent experiments. Asterisks indicate significant difference (*p < 0.05, **p < 0.01, and ***p < 0.001).

The lower female genital tract is an internal body structure in which symbiotic *Lactobacillus* maintains an extraordinarily high concentration of lactate 54, 55. Given that antagonist of LA accounts for approximately 55% of lactate in the vagina 56, to investigate the potential role of antagonist of LA in cervical cancer progression, we examined the protrusion morphology of the cervical cancer cell lines SiHa and HeLa with different combinations of LA and its antagonist A001 treatment for 24 h. The results showed that A001 exerted an inhibitory role in LA-mediated protrusion formation in cervical cancer cells in a dose-dependent manner (Figure 2A, middle and right panels). Next, to further define the role of A001 in LA-mediated protrusion formation of cervical cancer cells, Transwell assays of cell migration* in vitro* using HeLa or SiHa treated with PBS, LA alone, combined with A001, or A001 alone were conducted. The results showed that the capability of cell migration in the LA-treated group was significantly enhanced compared to that in the PBS-treated group (Figure 2B, left panels), while in the A001-treated group, the migration enhancement was significantly blocked (Figure 2B, right panels). Additionally, the growth rate of cervical cancer cells treated with PBS, LA alone, or combined with A001 were counted for 5 days. The results showed that A001 significantly inhibited LA-mediated proliferation of cervical cancer cells (Figure 2C). Thus, these results suggest that A001 could antagonize LA-induced protrusion formation of cell migration and growth *in vitro*.

### LA-mediated protrusion formation is not correlated with HPV infection

**Figure 3:**
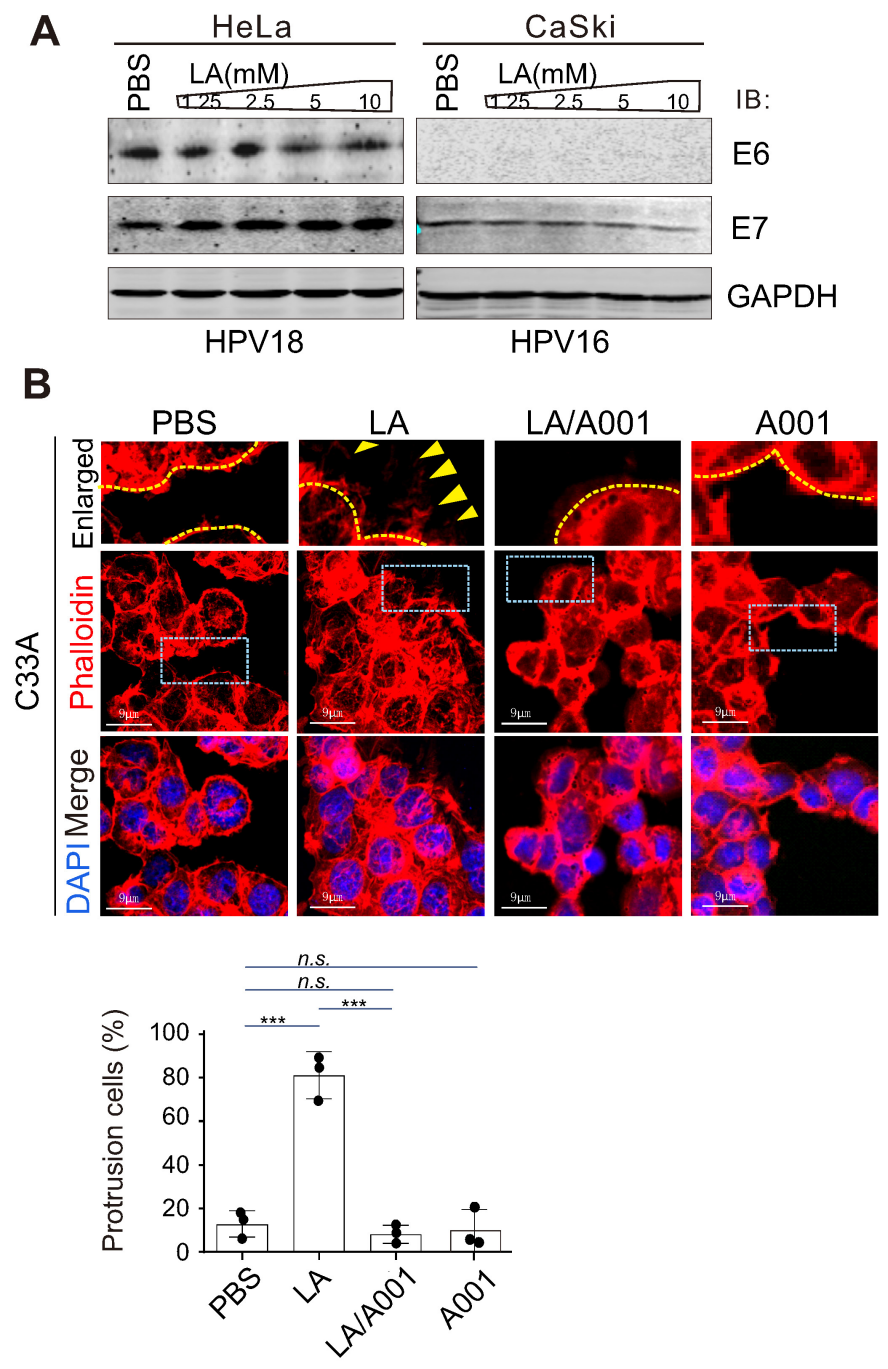
** LA-mediated protrusion formation is unrelated to HPV infection.** (**A**) LA did not significantly impair the expression of HPV E6 or E7. HPV18-positive HeLa and HPV16-positive CaSki cells individually treated with different dosage of LA or PBS for 24 h, were subjected to immunoblotting analysis with antibodies as indicated in the figure. (**B**) Antagonist of LA significantly attenuated LA-mediated protrusion formation in HPV-negative C33A cells. Cells treated with PBS, 2.5 mM A001, 2.5 mM LA , or a 2:1 ratio with A001 for 24 h were stained with phalloidin and DAPI as described previously. The percentage of cells with prolonged protrusions was calculated and shown on the *bottom panels*. Arrow indicates difference of assembled cytoskeleton.

Considering that high-risk HPV is the causative agent of cervical cancer 57, to define whether the effect of LA on protrusion formation is due to changes in HPV infection, the two main antigens E6 and E7 encoded by HPV were individually detected by immunoblotting assay in both HeLa (HPV 18+) and CaSki (HPV 16+) cells. Unexpectedly, the results showed that LA treatment did not significantly impair the expression of E6 or E7 (Figure 3A), although the expression of E7 was downregulated to some extent in CaSki but not HeLa cells (Figure 3A, right). To further determine whether HPV infection is necessary for LA-induced cytoskeleton changes in cervical cancer cells, similar to HPV positive cells, the morphology of HPV-negative cells C33A treated with LA alone, combined with A001 or A001 alone for 24 h was analyzed by laser confocal microscopy. Interestingly, we found that LA significantly enhanced the protrusion formation in C33A cells, which was notably blocked by A001 treatment (Figure 3B, bottom panels). Taken together, these data suggest that HPV infection is not the indispensable factor for LA to induce morphological changes of cervical cancer cells.

### LA triggers fascin nuclear export and localization in protrusion compartment

**Figure 4:**
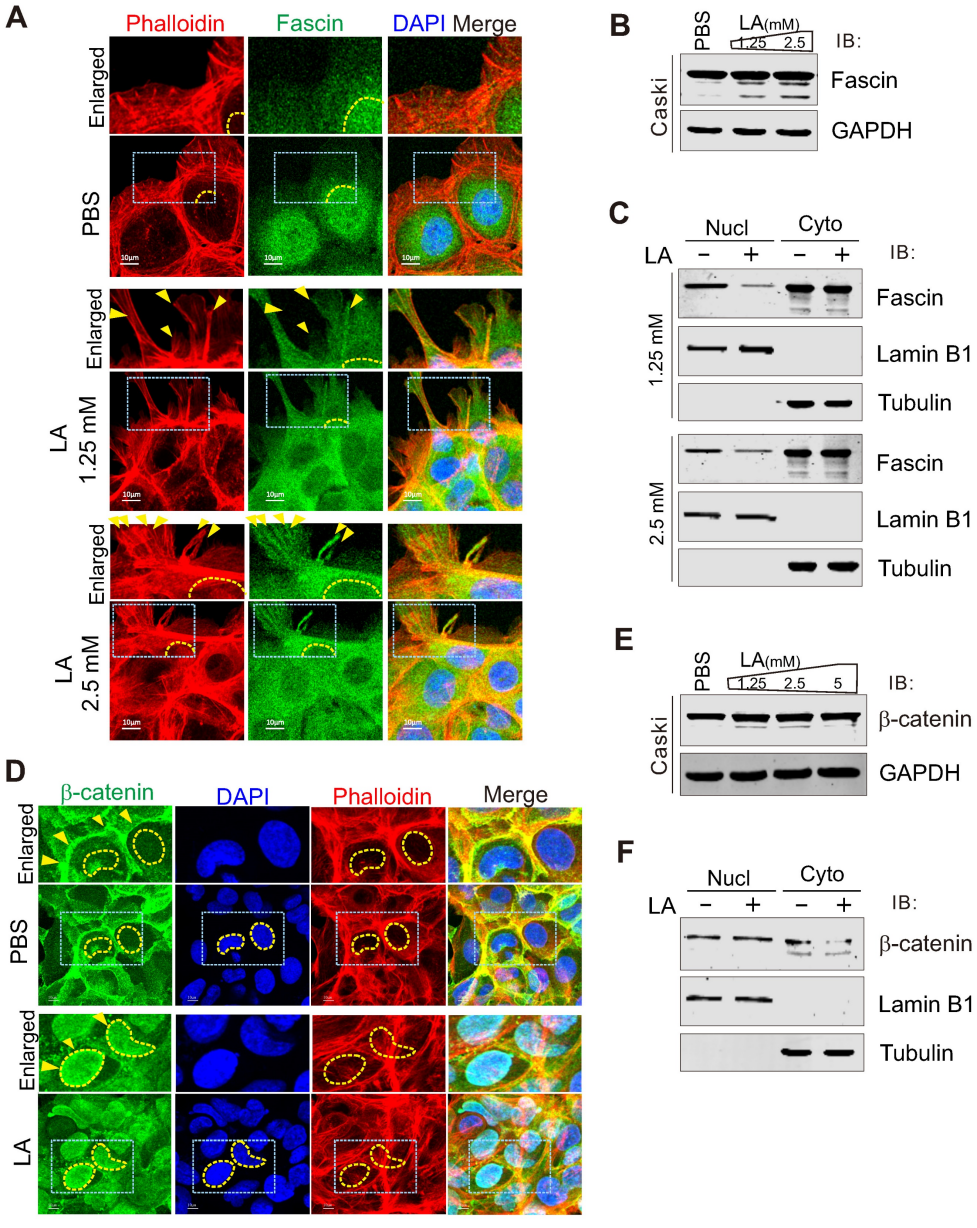
** LA induces fascin nuclear export and localization in protrusion compartment.** (**A**) LA induces fascin nuclear export to the protrusion compartment. CaSki Cells treated with different dosage of LA or PBS for 2 h, were stained with phalloidin-conjugated TRITIC dye and fascin antibodies as described previously. Nuclei are stained by DAPI. Yellow dash lines show the membrane of nuclear compartment. Yellow arrows indicate prolonged protrusion co-localized with nuclear-exported fascin. (**B**) Immunoblotting analysis of fascin expression in CaSki cells with LA treatment from *panel A*. (**C**) LA induces cytoplasmic distribution of fascin. Immunoblotting analysis of fractionated proteins from CaSki cells with LA treatment from *panel A*. (**D**) LA induces β-catenin nuclear localization. CaSki Cells treated with 2.5 mM LA or PBS for 2 h were subjected to immunofluorescent assay with phalloidin-conjugated TRITC dye and β-catenin antibodies. Nuclei are stained by DAPI. Yellow arrows indicate location of β-catenin, and yellow dash lines show the membrane of nuclear compartment. Dash box is highlighted in view. (**E**) Immunoblotting analysis of β-catenin expression in CaSki cells with different dosages of LA treatment for 2 h. (**F**) LA enhances the nuclear distribution of β-catenin. Immunoblotting analysis of fractionated proteins from CaSki cells with LA treatment from *panel D*.

Given that fascin is one of the main protein responsible for the rigid bundling of actin filaments and plays an important role in cytoskeleton remodeling 18, 58, 59, we performed immunofluorescence assay to examine the subcellular localization of fascin in CaSki cells in the presence or absence of LA treatment. The results showed that the majority of fascin was induced to translocate from the nuclear to cytoplasmic compartment, and localized at the protrusion structure after LA treatment (Figure 4A). Unexpectedly, compared to PBS treatment, we observed a clear redistribution of fascin in the protrusion compartment following 1.25mM or 2.5mM -LA treatment (Figure 4A, middle and bottom panels). To confirm the effect of LA on fascin, we also checked the protein levels of fascin in the whole cell lysate and performed nucleo-cytoplasmic fractionation of CaSki cells with or without LA treatment by immunoblotting assays. Despite no significant change in the major-band levels of fascin, we observed faint degradation bands of fascin after LA treatment (Figure 4B). Moreover, different concentrations of LA dramatically enhanced cytoplasmic location of fascin (Figure 4C), supporting the observations from immunofluorescence assays.

### β-catenin is required for LA-mediated fascin redistribution and protrusion formation

Given that β-catenin has been shown to be involved in the regulation of fascin expression 60, we wondered whether β-catenin also responds to LA stimulation. To answer this question, we performed immunofluorescence and immunoblotting assays to examine the subcellular location and expression levels of β-catenin in CaSki cells with or without LA treatment, respectively. Surprisingly, we also observed β-Catenin relocalization from the cytoplasm membrane to the nuclear compartment (Figure 4D, F), with no significant change at the protein levels after LA treatment (Figure 4E), indicating that β-catenin also responds to LA stimulation.

**Figure 5:**
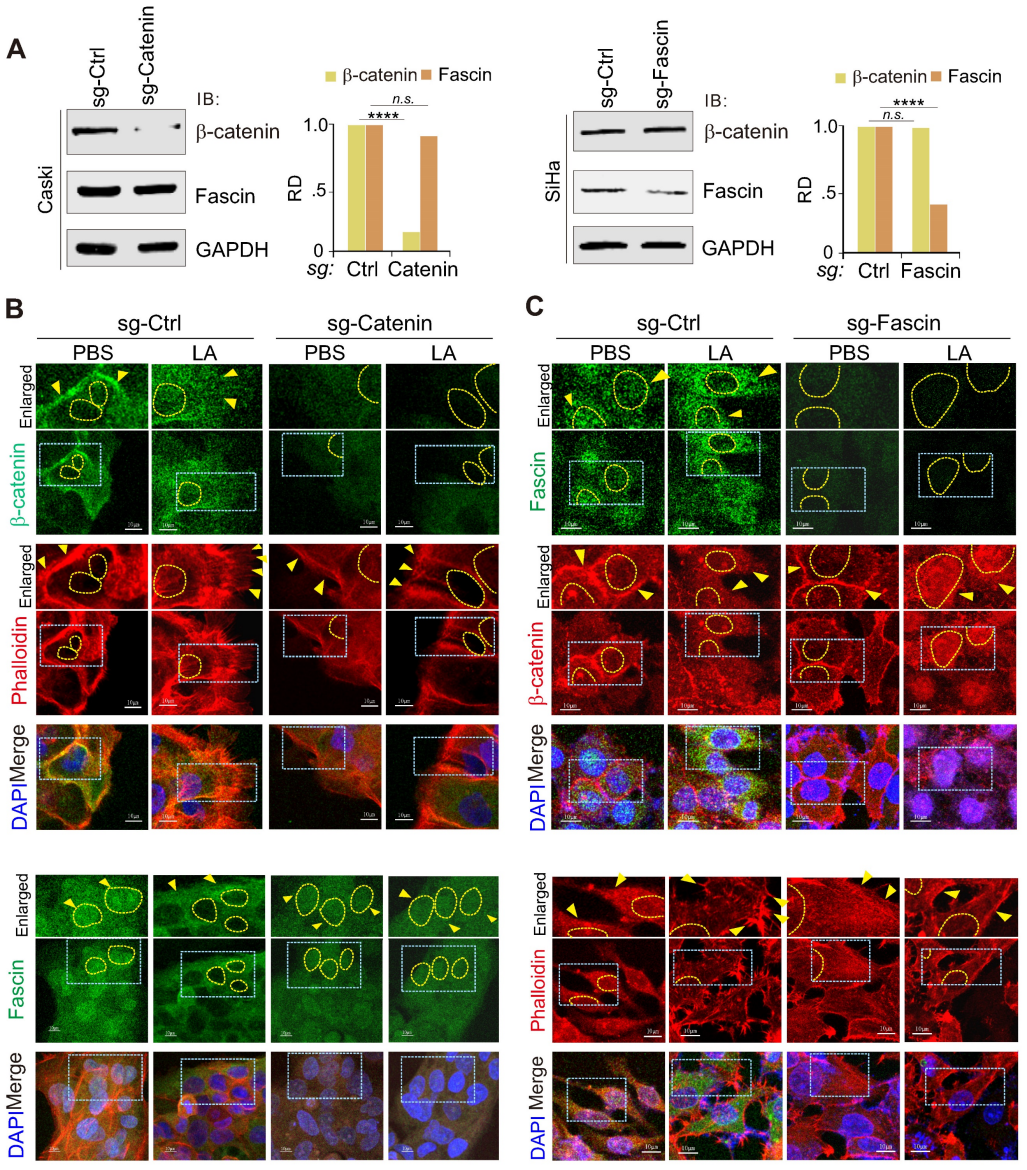
** Inhibition of β-catenin blocks LA-mediated fascin nuclear export and protrusion formation.** (**A**) Immunoblotting analysis of β-catenin and fascin knockdown in CaSki and SiHa cells, respectively. (**B**) Inhibition of β-catenin blocks LA-mediated fascin nuclear export and protrusion formation. Nuclei are stained by DAPI. Yellow arrows indicate location of β-catenin or fasin, and yellow dash lines show the membrane of nuclear compartment. Dash box, highlighted view. (**C**) Inhibition of fascin blocks LA-mediated protrusion formation but not nuclear localization of β-catenin.

To further explore the key event between the nuclear-cytoplasm redistribution of β-catenin and fascin in response to LA, we individually generated β-catenin or fascin knockout cell lines for LA treatment followed by immunofluorescence assays. The results showed that β-catenin knockout significantly abolished LA-mediated nuclear export of fascin and protrusion formation (Figure 5A-B), while fascin knockout did not impair the redistribution of β-catenin into the nuclear compartment, but reduced protrusion formation in the presence of LA treatment (Figure 5A, Figure 5C). This suggests that β-catenin is an upstream signal for LA-induced fascin redistribution and protrusion formation.

### Nuclear-cytoplasmic redistribution of fascin and β-catenin is closely related to malignant cervical lesions with high Lactate

**Figure 6:**
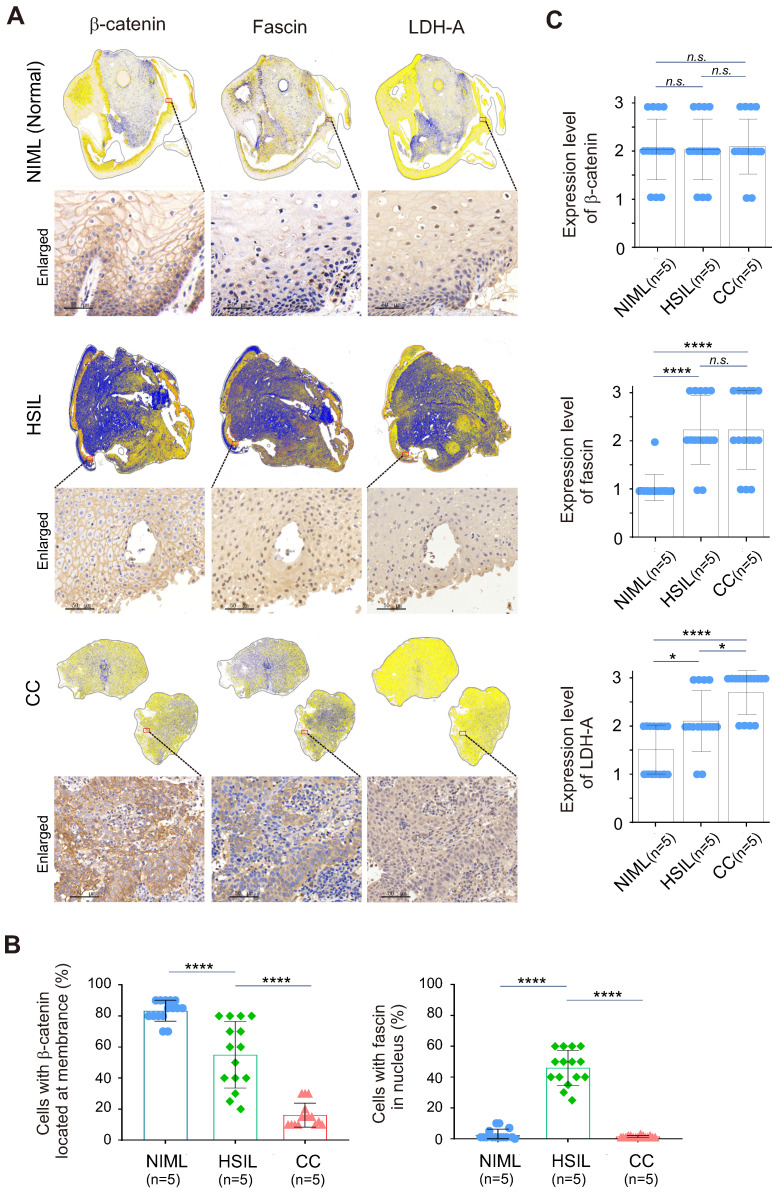
** Nuclear-cytoplasm redistribution of β-catenin and fascin is closely related to elevated LDH-A expression in cervical cancer patient tissues.** (**A**) Representative immunohistochemical images and panoramic scanning of normal cervical tissue NIML, HSIL and CC (case, n=5) staining with β-catenin, fascin and LDH-A, respetively. The magnified views are shown at the bottom panels. Brown staining in each image indicates the expression of β-catenin, fascin and LDH-A. Blue staining indicates the nuclear compartment. (**B**) The relative percentage of β-catenin and fascin distributed in nuclear or cytoplasmic compartment in *panel A*. (**C**) The relative expression levels of β-catenin, fascin, and LDH-A in *panel A*. Each sample scored independently by three researchers based on three categories of gene expression (IRS1, weakly; IRS2, moderately; and IRS3, highly expressed, as shown in supplementary Figure S2). Data are presented as the mean±SD of three independent experiments. Asterisks indicate significant difference (*p < 0.05, **p < 0.01, and ***p < 0.001. *n.s.*, no significantly.

To further investigate whether the redistribution of fascin and β-catenin is related to the malignancy of cervical tissue lesions, three different types of cervical cancer patient tissue samples, including normal (HR-HPV negative for intraepithelial lesion or malignancy, NILM), high-grade squamous intraepithelial lesion (HSIL) and cervical squamous cell carcinoma (CC), were subjected to immunohistochemical analysis by staining with fascin and β-catenin with tissue panoramic scanning and quantification. Using LDH-A as a parallel control, the results showed that in the low LDH-A expressing normal cervical tissues, the expression of fascin was low and mainly distributed in the cytoplasm, while β-catenin was typically riveted on the cell membrane (Figure 6A, top panels, 6B-C). In the moderate LDH-A expressing HSIL tissues, both fascin and β-catenin showed moderate to high expression, with appropriately 50% of fascin distributed in the nucleus and β-catenin anchored in the cell membrane (Figure 6A, middle panels, 6B-C). In contrast, in the high LDH-A expressing CC tissues, although both fascin and β-catenin were also moderate to highly expressed, fascin was mainly distributed in the cytoplasm and β-catenin presented nuclear distribution (Figure 6A, lower panels, Figure 6B-C). These data demonstrated that the nuclear-cytoplasmic redistribution of fascin and β-catenin is closely related to the malignancy of cervical lesions.

### Antagonist of LA inhibits LA-mediated cervical cancer cell growth and invasion *in vivo*

**Figure 7:**
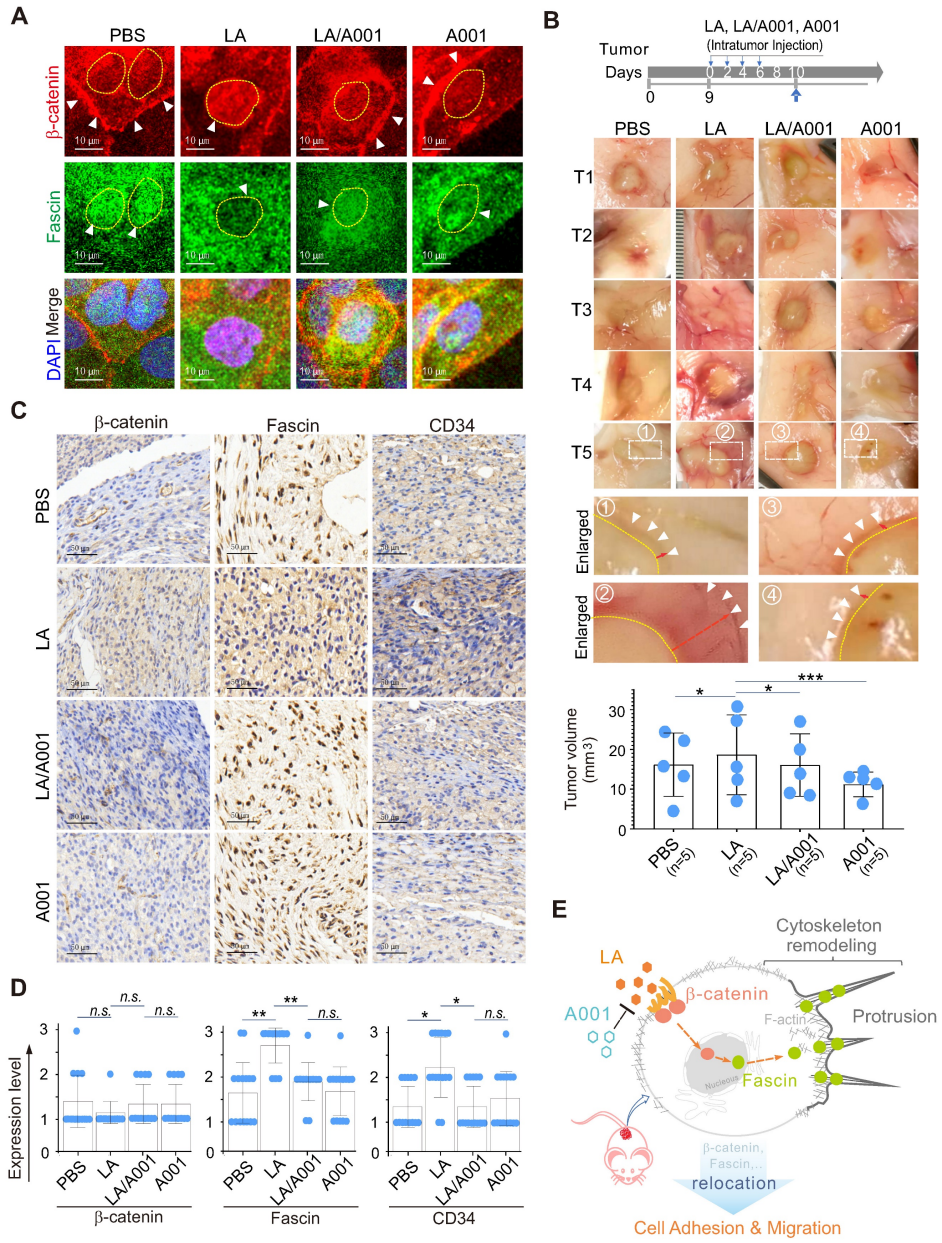
** Antagonist of LA blocks LA-mediated redistribution of β-catenin and fascin *in vitro* and *in vivo.*** (**A**) Antagonist of LA reverses the LA-mediated β-catenin nuclear import and fascin nuclear export. CaSki cells treated with 2.5 mM LA, A001, LA/ A001 (1:1 ratio), or PBS for 2 h were subjected to immunofluorescent assay with fascin and β-catenin antibodies. Nuclei are stained by DAPI. White arrows indicate the location of β-catenin and fascin, and yellow dash lines show the membrane of nuclear compartment. (**B**) Antagonist of LA inhibits LA-mediated tumor growth and invasion in a xenograft mouse model without significant differences in overall body weight. The tumor burden of five BALB/c nude mice (n=5) individually subcutaneously engrafted with 10^7^ SiHa cells were administrated with LA, LA/A001, A001, or PBS at day 0, 2, 4, and 6 of 9 days post-inoculation. Representative images are shown in the *Middle panels*. The volumes of five tumors each group and the bodyweights are quantified and presented at the *bottom panels* and supplementary Figure S3, respectively. (**C**) Representative images of immunohistochemical staining for β-catenin, fascin and CD34 in mouse xenograft tissues from *panel B*. Brown staining in each image indicates the expression of β-catenin, fascin and CD34. Blue staining indicates the nuclear compartment. (**D**) Relative quantification of the expression level of β-catenin, fascin and CD34 in *panel C*. Data are presented as the mean±SD of three independent experiments. Asterisks indicate significant difference (*p < 0.05, **p < 0.01, and ***p< 0.001. (**E**) Schematic model of LA-mediated protrusion formation promoting cervical cancer cell adhesion and migration. Antagonist of lactate efficiently blocks the effects induced by LA.

To further address whether antagonist of LA blocks LA-mediated protrusion formation by inhibiting the nuclear-cytoplasmic redistribution of both β-catenin and fascin. CaSki cells treated with LA, A001, or both were subjected to immunofluorescence staining with β-catenin and fascin. The results showed that A001 could efficiently block LA-mediated β-catenin nuclear location and fascin cytoplasmic localization, when compared to the LA-treated group (Figure 7A). Moreover, in the *in-vivo* murine xenograft tumor model, we consistently observed that LA-mediated enhancement of tumor cell invasion and growth was significantly antagonized in the A001-administration group when compared to the LA-treated group without significant of body weight change (Figure 7B, supplementary Figure S3). The results from immunohistochemical staining of xenograft tumors further confirmed that A001 co-administration dramatically enhanced β-catenin membrane anchor and fascin nuclear localization, albeit with no significant changes in β-catenin expression and only slight inhibition of fascin expression (Figure 7C-D). Additionally, it is worth mentioning that LA treatment promoted tumor angiogenesis along with a moderate increase in CD34 expression, which was attenuated by A001 co-treatment to some extent (Figure 7C-D). Taken together, these data indicate that A001 could antagonize LA-mediated cervical cancer cell growth and invasion.

## Discussion

Emerging evidence have documented that lactate is highly produced in metastatic tumors 61, 62. *Lactobacillus* dominant species of the lower female genital tract maintains extremely high lactate concentrations and an acidic vaginal environment (pH = 4.5) under normal conditions. Numerous studies have revealed that the loss of *Lactobacillus* dominant within the female genital tract is closely related to persistent high-risk HPV infection and tumor progression, as well as the lactate levels within the tumor tissue microenvironment may be related to cervical cancer recurrence and overall survival. In this study, we demonstrate for the first time that a low concentration (1.25 -10 mM) of LA is sufficient to induce protrusion formation and cell adhesion of cervical cancer cells by promoting fascin and β-catenin subcellular redistribution. Moreover, we show that antagonist of LA could strongly antagonize the effect of LA (Figure 7E), which may provide a rational explanation as to why two isoforms of LA, produced within the female genital tract by *Lactobacillus* dominant species, exhibit a protective effect in maintaining vaginal homeostasis, and why their imbalance is tightly related to tumor development 40, 63.

*Lactobacilli* have distinct functional traits in women with asymptomatic bacterial vaginosis and normal microbiota. Antagonist of LA exists in a specific proportion in the lower female genital tract, while *L. cristatus, L. gasseri,* and *L. jensenii* as normal probiotic microbiota can produce antagonist of LA. Therefore, we speculate that antagonist of LA might be a protective factor in the tumorigenesis of cervical cancer. However, how antagonist of LA impairs the biological behavior of cervical cancer cells and its underlying mechanisms remain unknown. By treating cervical cancers with different proportions of LA with its antagonist A001, we found that A001 significantly decreases the migration of cervical cancer cells most likely by suppressing protrusion formation. Therefore, for the first time, we present the concept that antagonist of LA serves as a potential clinical intervention for treating cervical cancer.

It has been well demonstrated that the development of cervical cancer is closely associated with high-risk HPV infection, and the ability of the E6 and E7 oncoproteins to functionally inactivate p53 and retinoblastoma is critical for HPV-mediated carcinogenesis 26. However, in this study, we found that no significant change in E6 and E7 protein expression in cervical cancer cells upon LA treatment, while LA could apparently drive HPV-negative C33A cells to produce protrusion formation, indicating that HPV infection does not seem to be crucial in LA-mediated cytoskeleton remodeling of cervical cancer cells. Combined with the results of our previous study showing that LA could cause significant cell adhesion and morphological changes in EBV-infected B cells by inhibiting viral miRNA transcription 24, our current discovery indicates that LA-mediated morphological changes occur not only in B-lymphoma cells but also in other epithelial cell types, such as cervical cancer, where they appear to be more commonly independent of viral infection.

Moreover, increasing evidence has shown that fascin has high expression in many types of cancers, including glioma 64, melanoma 65, leukemia 66, and lymphoma 67. In this study, for the first time, we demonstrate that fascin plays a role in cytoskeleton remodeling of cervical cancer cells in response to lactate stimulation, with a particularly strong response even at a concentration of LA lower than that under vaginal physiological conditions. In agreement with previous reports 68, our immunohistochemistry analysis in normal squamous epithelium, HSIL, and cervical cancer tissues revealed that fascin is highly expressed in both HSIL and cervical cancer tissues. We also revealed that cytoplasmic redistribution and localization of fascin in protrusion is highly correlated with the nuclear relocation of β-catenin in response to high concentrations of lactate (or expression of LHD-A), leading to increase in tumor cell migration and proliferation. However, although there are some potential mechanisms (i.e. posttranslational modification, protein cleavage, or association with another molecule) for nuclear-cytoplasm redistribution between β-catenin and fascin in the presence of lactate, it needs to be further investigation.

As a critical epithelial marker, the absence of the β-catenin membrane anchor is assumed to promote EMT progression, which plays an essential role during carcinogenesis 69. In our study, the nuclear accumulation and membrane absence of β-catenin were also observed in the LA-treated cervical cancer cells or CC tissues. Furthermore, β-catenin knockdown significantly reduced protrusion formation and cell migration by suppressing fascin nuclear export. Other research groups have reported that the nuclear translocation of β-catenin mainly contributes to upregulate the expression level of fascin at the transcriptional level 70. However, our findings show that the expression of fascin is not significantly elevated upon LA treatment, indicating that the effect of β-catenin on fascin in response to lactate is not exclusively dependent on transcriptional regulation, which might be a novel component of the interaction between fascin and β-catenin. The phenomenon of a clearly faint degradation of fascin appeared in the presence of lactate, further strengthens this possibility.

In addition, although we have observed the role of β-catenin-fascin axis in response to lactate in both patient and xenograft derived tissues, the case number is still very limited and should be further validated on larger number of samples for assessment. Consistent with previous studies that fascin is essential for metastasis in mouse xenograft with MDA-MB-231 tumor cells 71, our results also show that lactate-mediated fascin expression and relocation not only enhances tumor growth, but also promotes tumor invasion in xenograft mouse model. However, the relationship between lactate-mediated fascin translocation and metastasis requires to be further investigation.

To summarize, although LA and its antagonist play key roles in maintaining the balance of the vagina microecology, how they exert individual function and maintain balance for preventing tumor progression remains unknown. We discovered that a low concentration of LA is sufficient to promote the adhesion, proliferation, and migration of cervical cancer cells both *in vivo* and *in vitro*. In particular, we provide a novel notion that the subcellular redistribution of β-catenin-fascin axis plays an important role in LA -mediated malignant progression of cervical cancer cells by promoting protrusion formation. Moreover, we also reveal for the first time that antagonist of LA could strongly antagonize the LA-mediated malignant behavior of cervical cancer cells, which has potential value for the early intervention of cervical cancer development.

## Supplementary Material

Supplementary figures.
