# Supplementary material for: Lactate-mediated Fascin protrusions promote cell adhesion and migration in cervical cancer
Source: Theranostics. 2023 Apr 17;13(7):2368–83. doi: 10.7150/thno.83938 (PMC10157738; doi:10.7150/thno.83938)
Supplement: Supplementary file 1 — Supplementary figures. [file thnov13p2368s1.pdf]

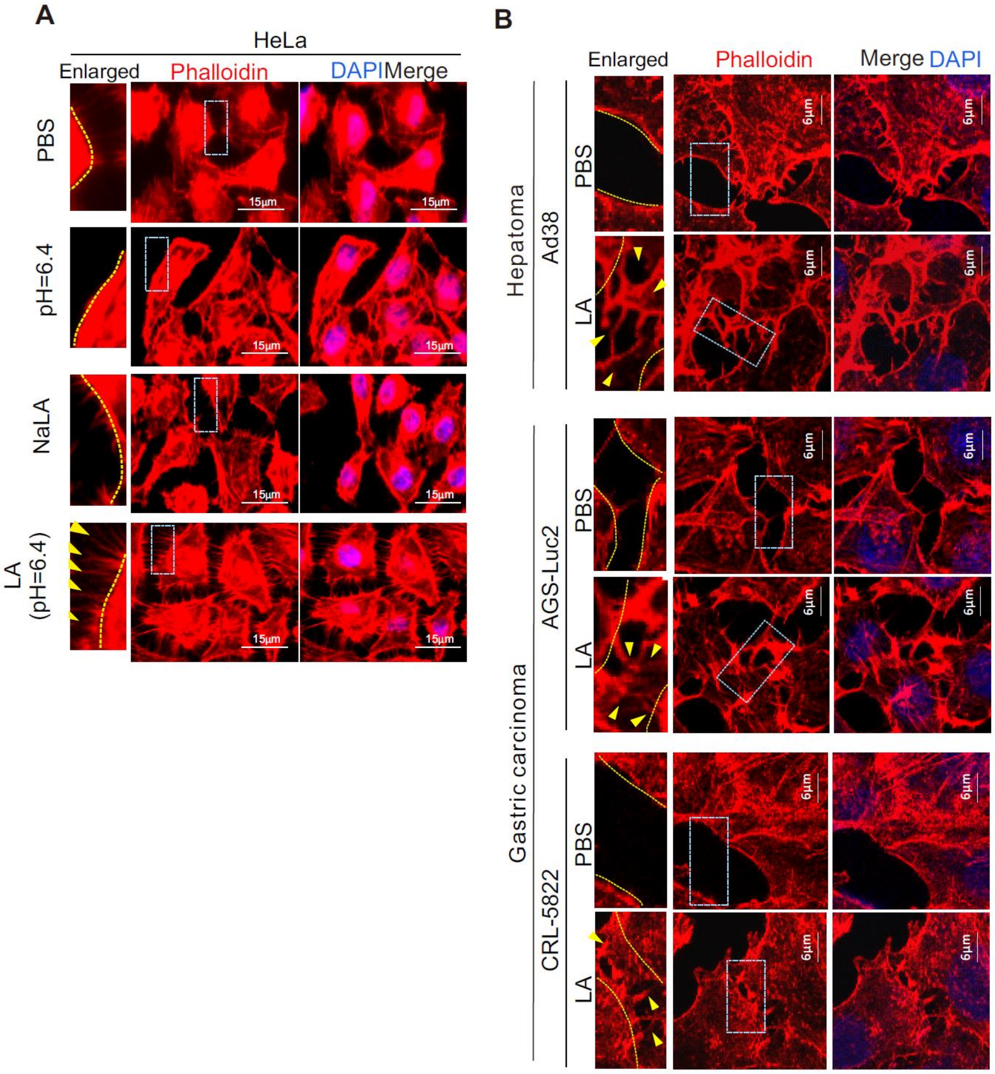

Figure S1 LA enhances protrusion formation in various types of cancer cells. Related to Figure 1.

(**A**) Lactic acid (LA) induces morphological changes in HeLa cells. Representative EVOS immunofluorescence microscopy photographs of cell morphology after treatment with 10 mM LA, LA-Na, PBS or medium at pH=6.4 for 24 h are shown. Cells were stained with phalloidin conjugated with TRITC. Nuclei are stained by DAPI. Yellow arrows in enlarged image indicates prolonged protrusions. Similar observation in CaSki cells with treatment was not shown. (**B**) Representative microscopic photographs of hepatoma and gastric carcinoma cells with or without LA treatment as indicated in panel A.

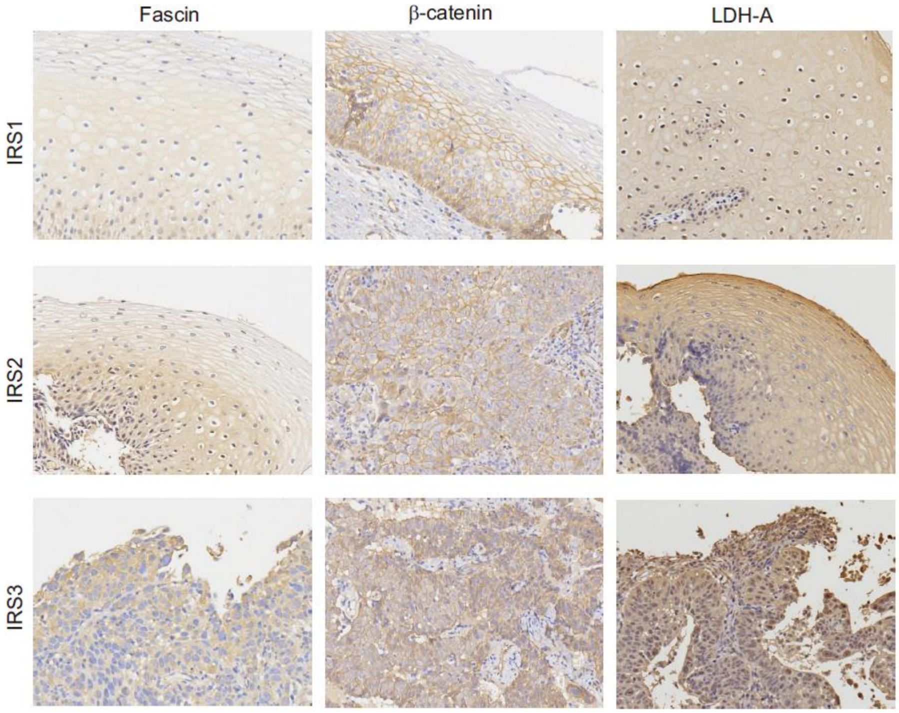

Figure S2 Representative microphotographs of IRS score for fascin, β-catenin and LDH-A. Related to Figure 6.

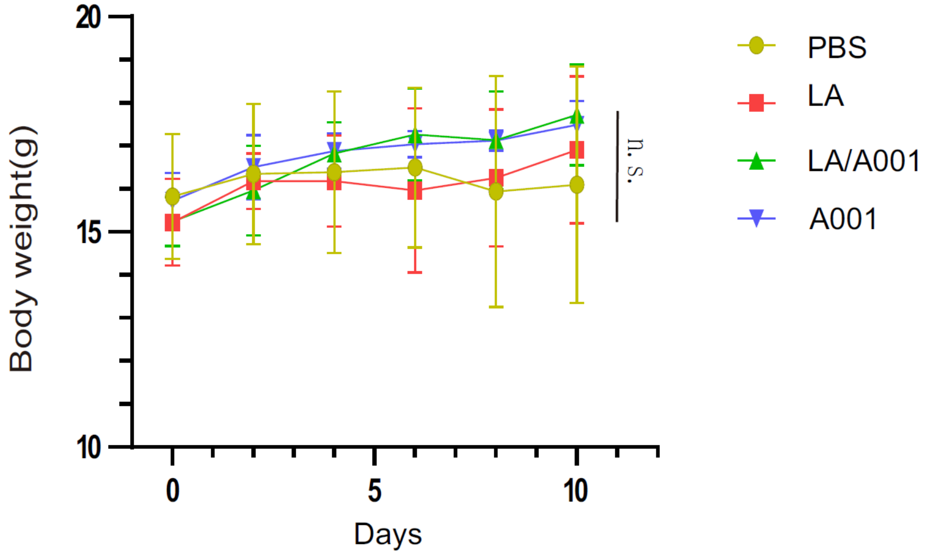

Figure S3 Body weight of mice with administration in a xenograft model. Related to Figure 7.
The tumor burden of five BALB/c nude mice (n=5) individually subcutaneously engrafted with 10^7 SiHa cells were administrated with LA, LA/A001, A001, or PBS at day 0, 2, 4, and 6 of 9 days post-inoculation as described in methods section and indicated in the figure 7B.
